# Supplementary material for: Temporal clustering of disorder events during the COVID-19 pandemic
Source: PLoS One. 2021 Apr 22;16(4):e0250433. doi: 10.1371/journal.pone.0250433 (PMC8062074; doi:10.1371/journal.pone.0250433)
Supplement: S1 File — (PDF) [file pone.0250433.s001.pdf]

Supporting Information:
Temporal Clustering of Disorder Events During the COVID-19 Pandemic

Gian Maria Campedelli[1* ‡], and Maria R. D'Orsogna[2,3]

1 Department of Sociology and Social Research, University of Trento, Trento, Italy
2 Department of Computational Medicine, University of California Los Angeles, Los Angeles, CA, United States of America
3 Department of Mathematics, California State University, Northridge, Los Angeles, CA, United States of America

∗ Corresponding author
E-mail: gianmaria.campedelli@unitn.it

S1 File

S1.1 ACLED event types

In this Appendix we illustrate the various categories the ACLED codebook uses to classify disorder events [1]:

Violence against civilians involve one organized armed group deliberately inflicting violence against unarmed non-combatants. Perpetrators of violent acts can include state forces and affiliates, rebels, militias or other marginal subjects. Attempts to inflicting harm are also included, such as attempted kidnappings.

Riots are characterized by demonstrators or mobs engaging in violent, disruptive actions such as property destruction. Riots can emerge from peaceful protests and are generally characterized by the use of unsophisticated weapons.

Protests refer to public demonstrations involving participants that do not engage in violent activity, although violence may be used against them. Symbolic acts such as publicly displaying flags are not coded as protests if they are not accompanied by a demonstration. Parliamentary walkouts and/or individual acts such as self-harming are not included.

Battles involve violent interactions between politically organized armed groups at a particular time and location. At least two armed actors must be present; these may be armed and may include state, non-state and external entities. There is no minimum threshold for the number of fatalities.

S1.2 k-means clustering

The purpose of k-means clustering is to partition a set of n points $\{x_1, \cdots, x_n\}$ into k clusters $C_1, \cdots, C_k$ [2]. This iterative algorithm seeks to identify clusters C_i by considering their centroids ν_i and by minimizing the average distance of the data points within it to the centroid. Therefore, the k-means algorithm tries to find $\mathbf{C} = \{C_1, \cdots, C_k\}$ and ν_i defined as

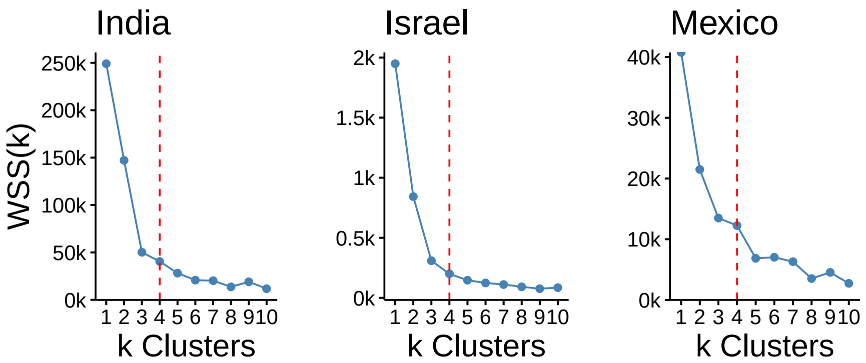

Fig S1. From left to right: k-means clustering applied to Israel, India, Mexico. On the vertical axis is the WSS(k). Note that the scales reflect the spatial extent of the countries. India being the largest by territorial extent is associated to the largest WSS(k) range, India being the smallest is associated to the smallest WSS(k) range. The vertical line denotes our elbow method best estimate for the optimal k^* value which we identify as $k^* = 4$ in all countries.

$$\arg\min_{\mathbf{C}} \sum_{i=1}^{k} \sum_{x \in C_i} \|x - \nu_i\|^2 \tag{S1}$$

Here, $\|x - \nu_i\|^2$ is the square of the Euclidean distance between the points in a given cluster and its centroid ν_i. Procedurally, k centroids ν_i are initialized and each data point is assigned to its closest centroid. The mean of the positions of all points within a cluster define the new centroid. An iterative process ensues until discrepancies between iterations falls below a given threshold.

S1.2.1 Finding the optimal number of k

To identify the optimal number of clusters k^* we utilized the heuristic elbow method. Here, k-means clustering is applied for several increasing values of k. Once clusters are identified, the sums of the square of the distance of each point within a cluster to its centroid is calculated. This k-dependent quantity is termed WSS(k), within-cluster sum. As k increases, more clusters are possible, hence, one may expect the WSS(k) to decrease as a function of k as there may be a centroid closer to them. However, beyond a critical value k^* the decrease may be marginal, indicating that allowing for extra clusters does not improve on the compactness of the clustering process. The value of k^* beyond which decreases in WSS asymptote yields the elbow, optimal value of k^*. In our work we use $1 < k < 10$; as can be seen from for all three countries of interest, India, Israel and Mexico, the optimal k^* value is $k^* = 4$.

S1.3 Hawkes Process parameter estimation

We use MLE to derive the Hawkes process parameters μ, α, β. These emerge as the ones that maximize the loglikelihood function defined as

$$\log L(\mu,\alpha,\beta|t_1,...,t_n) = \sum_{i=1}^{n} \log(\lambda(t_i)) - \int_0^{t_n} \lambda(t)dt \tag{S2}$$
$$= \sum_{i=1}^{n} \log\left[\mu + \alpha \sum_{j=1}^{i-1} \mathrm{e}^{-\beta(t_i - t_j)}\right] - \mu t_n + \frac{\alpha}{\beta} \sum_{i=1}^{n} \left[\mathrm{e}^{-\beta(t_k - t_i)} - 1\right],$$

where $\{t_1, ..., t_n\}$ is the set of the times of occurrence of given events. The loglikelihood function compares the value of the intensity function of the Hawkes process $\lambda(t)$ at event times $\{t_1, ..., t_n\}$ to the cumulative value of the function within the continuous interval $0 \leq t \leq t_n$. Maximizing the loglikelihood function yields parameters which best represent the actual event data. In this work we maximize $\log L$ through the Nelder-Mead approach as available in the `ptproc` package in R [3].

S1.4 Event Distribution - Cluster wise

S1.4.1 India

Distribution summaries are shown in Fig. S2: C2 has the highest average number of disorders per week and the highest variability, followed by C1. Interestingly, while C4 has the second-lowest average number of disorders, it exhibits outliers, coinciding with week $j = 19$ (51 events) and week $j = 24$ (60 events).

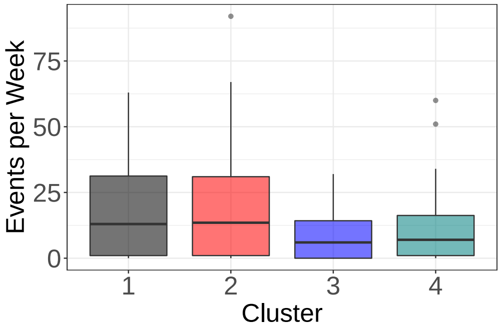

Fig S2. Cluster-wise boxplot of disorder events in India. The most occurrences arise in clusters C1, C2, where the most densely populated states are located. C4 displays several outliers.

S1.4.2 Israel

Figure S3 reveals low values of averaged weekly disorders, however many outliers emerge corresponding to the interval between weeks $j = 37$ and $j = 50$ mentioned above.

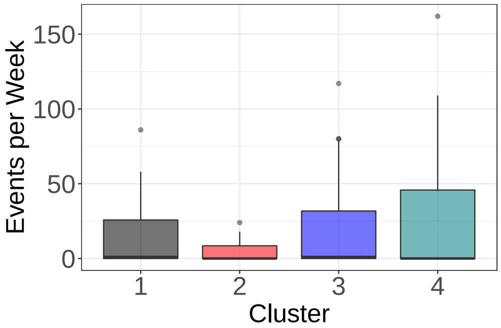

Fig S3. Cluster-wise boxplot of disorder events in Israel. The most occurrences arise in clusters C1, C3 and C4 where the major cities of Haifa, Tel Aviv and Jerusalem are located.

S1.4.3 Mexico

Figure S4 summarizes the distribution of events in Mexico at the weekly level. As mentioned, C4 has the highest average and variability in event counts, followed by C2, whereas in C3 and C1 fewer events are recorded. Interestingly, C1 is characterized by a very low variability. Thus, while spikes in activity and fluctuations emerge in other clusters, events in C1 are more uniformly distributed.

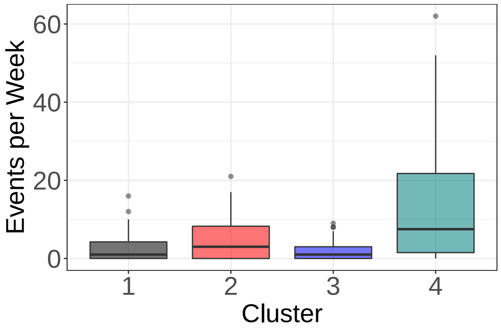

Fig S4. Cluster-wise boxplot of disorder events in Mexico. The most occurrences arise in cluster C4, where the most populous and dense areas of Mexico City and Mexico state are located.

S1.5 Cluster-based analysis: Pearson's correlation coefficients

In this section we list the numerical values of the Pearson coefficient r correlating the number of weekly of events in pairs of clusters within a given country. If we denote two clusters within a country C_X and C_Y then r is defined as

$$r = \frac{\mathrm{E}(X - \mu_x)E(Y - \mu_y)}{\sigma_X \sigma_Y} \tag{S3}$$

where X, Y are the sets of weekly data in clusters C_X and C_Y, respectively, μ_X, μ_Y their averages, and σ_X, σ_Y their standard deviations. Pearson's correlation coefficient ranges from -1 to 1; $r = 1$ implies a perfect, positive, linear relationship between the

two datasets whereas $r = -1$ implies a perfect negative one. As $|r|$ decreases, correlations become weaker, so that $r = 0$ implies data points in the two sets X, Y are not correlated. In our work, X, Y are the either the sets of weekly events $\{n_j^X\}, \{n_j^Y\}$ in each cluster or the sets of differentiated weekly events $\{\Delta n_j^X\}, \{\Delta n_j^Y\}$ where $\Delta n_j^X = n_j^X - n_{j-1}^X$ and $\Delta n_j^Y = n_j^Y - n_{j-1}^Y$. Below we show how these quantities manifest in each of the three countries under investigation.

S1.5.1 India

	C1	C2	C3	C4
C1	1.000			
C2	0.678	1.000		
C3	0.724	0.595	1.000	
C4	0.598	0.644	0.658	1.000

	C1	C2	C3	C4
C1	1.000			
C2	0.124	1.000		
C3	0.219	0.301	1.000	
C4	0.450	0.339	0.512	1.000

Table 1. Pearson's correlation matrices for India and shown in Fig. 6. Top: Entries represent correlation coefficients r derived on weekly events $\{n_j\}$ for the period January 3rd to December 12th 2020 and between the associated clusters. Overall, correlation values are moderately large and uniform. The highest $r = 0.724$ is observed between clusters C1 and C3. Bottom: Entries represent correlation coefficients r derived on differentiated weekly events $\{\Delta n_j\}$ and show much weaker correlation, implying a reduced synchrony in the rate of change of the occurrence of events.

S1.5.2 Israel

	C1	C2	C3	C4
C1	1.000			
C2	0.996	1.000		
C3	0.998	0.995	1.000	
C4	0.998	0.995	0.999	1.000

	C1	C2	C3	C4
C1	1.000			
C2	0.972	1.000		
C3	0.986	0.954	1.000	
C4	0.986	0.958	0.995	1.000

Table 2. Pearson's correlation matrices for Israel and shown in Fig. 10. Top: Entries represents correlation coefficients r derived on weekly events $\{n_j\}$ for the period January 3rd to December 12th 2020 and between the associated clusters. Correlation values approach unity, revealing large synchrony within the country. Bottom: Entries represent correlation coefficients r derived on differentiated weekly events $\{\Delta n_j\}$. These remain very large, confirming the large degree of synchrony in the rate of change of events in the country.

S1.5.3 Mexico

	C1	C2	C3	C4
C1	1.000			
C2	0.675	1.000		
C3	0.632	0.571	1.000	
C4	0.753	0.826	0.772	1.000

	C1	C2	C3	C4
C1	1.000			
C2	-0.092	1.000		
C3	0.203	-0.445	1.000	
C4	0.286	0.140	0.246	1.000

Table 3. Pearson's correlation matrices for Mexico and shown in Fig. 14. Top: Entries represents correlation coefficients r derived on weekly events $\{n_j\}$ for the period January 3rd to December 12th 2020 and between the associated clusters. Overall, correlation values are moderately large. The highest $r = 0.826$ is observed between the geographically contiguous clusters C2 and C4. The lowest $r = 0.571$ is observed between clusters C2 and C4. Bottom: Entries represent correlation coefficients r derived on differentiated weekly events $\{\Delta n_j\}$ show vanishing or even negative correlation and implying lack of synchrony in the rate of change of the occurrence of events.

S1.6 Hawkes process in a restricted time window

In this section we apply the Hawkes process to disorder events recorded from the CDT from January 3rd to October 10th 2020. Similarly to what observed for the entire data set, the Hawkes process outperforms the Poisson process in all three countries and in all clusters, even in this limited time range. A noteworthy observation is that while the sequence of events in C4 in Israel is appropriately described by a Hawkes process until October 10th 2020 as per Table 5, the sequence of events that extends to December 12th is not as per Table 4, confirming that disorders in Israel in Fall 2020 are even extremely clustered than what predicted by Hawkes processes.

S1.6.1 India

**Cluster**	**India (all)**	**India (C1)**	**India (C2)**	**India (C3)**	**India (C4)**
Number of events	2,744	852	946	408	538
μ	0.291	0.537	0.332	0.120	0.662
α	2.075	1.447	1.538	0.495	1.518
β	2.020	1.223	1.400	0.462	1.078
γ	0.973	0.845	0.910	0.933	0.710
$\mu/(1-\gamma)$	10.777	3.464	3.666	1.791	2.282
Hawkes AIC	-9230	-825	-1274	204	-89
Poisson AIC	-7360	-371	-526	428	195
KS Stat, D	0.147	0.097	0.103	0.154	0.063
KS Crit 95%, D_c^{95}	0.161	0.118	0.145	0.246	0.113
KS Crit 99%, D_c^{99}	0.193	0.141	0.174	0.295	0.135

Table 4. Statistical outcomes of the Hawkes process applied to data from India up to October 10th 2020. The Hawkes process outperforms the baseline Poisson process both nationwide and in each cluster, since the Hawkes AIC is always less than the Poisson AIC. The Hawkes process passes the KS test at the 95% significance level in all cases.

S1.6.2 Israel

Cluster	Israel (all)	Israel (C1)	Israel (C2)	Israel (C3)	Israel (C4)
Number of events	1,197	285	76	373	463
μ	0.341	0.207	0.640	0.184	0.081
α	20.927	10.383	6.865	11.159	13.901
β	19.749	8.987	5.368	10.107	13.626
γ	0.944	0.866	0.782	0.906	0.980
$\mu/(1-\gamma)$	6.089	1.544	2.935	1.957	4.050
Hawkes AIC	-7871	-742	-66	-1366	-2290
Poisson AIC	-1759	375	6	312	200
KS Stat, D	0.104	0.164	0.122	0.131	0.257
KS Crit 95%, D_c^{95}	0.164	0.207	0.375	0.224	0.355
KS Crit 99%, D_c^{99}	0.196	0.249	0.449	0.268	0.381

Table 5. Statistical outcomes of the Hawkes process applied to data from Israel up to October 10th 2020. The Hawkes process outperforms the baseline Poisson process both nationwide and in each cluster, since the Hawkes AIC is always less than the Poisson AIC. The Hawkes process passes the KS test at the 95% significance level in all cases.

S1.6.3 Mexico

Cluster	Mexico (all)	Mexico (C1)	Mexico (C2)	Mexico (C3)	Mexico (C4)
Number of events	1,193	135	254	91	703
μ	1.330	0.460	0.651	0.143	0.985
α	2.968	2.911	1.845	0.159	2.496
β	2.287	1.085	0.906	0.110	1.782
γ	0.771	0.373	0.491	0.695	0.714
$\mu/(1-\gamma)$	5.807	0.733	1.278	0.468	3.444
Hawkes AIC	-2337	318	325	315	-659
Poisson AIC	-1781	356	386	330	-324
KS Stat, D	0.036	0.065	0.071	0.134	0.029
KS Crit 95%, D_c^{95}	0.081	0.147	0.116	0.275	0.096
KS Crit 99%, D_c^{99}	0.097	0.176	0.139	0.330	0.115

Table 6. Statistical outcomes of the Hawkes process applied to data from Mexico up to October 10th 2020. The Hawkes process outperforms the baseline Poisson process both nationwide and in each cluster, since the Hawkes AIC is always less than the Poisson AIC. The Hawkes process passes the KS test at the 95% significance level in all cases.

References

1. ACLED. Armed Conflict Location & Event Data Project (ACLED) Codebook; 2019. Available from: `https://acleddata.com/acleddatanew/wp-content/uploads/dlm_uploads/2019/04/ACLED_Codebook_2019FINAL_pbl.pdf`.

2. Hastie T, Tibshirani R, Friedman J. The elements of statistical learning: Data mining, inference and prediction. 2nd ed. New York, NY: Springer Nature; 2013.

3. Peng RD. Multi-dimensional Point Process Models in R. UCLA; 2002. Available from: `https://escholarship.org/content/qt3n6609wb/qt3n6609wb.pdf?t=lnp7c3`.
